# Modeling current and potential distributions of mammal species using presence‐only data: A case study on British deer

**DOI:** 10.1002/ece3.5424

**Published:** 2019-07-11

**Authors:** Simon Croft, Alastair I. Ward, James N. Aegerter, Graham C. Smith

**Affiliations:** ^1^ National Wildlife Management Centre Animal and Plant Health Agency York UK; ^2^ Department of Biological and Marine Sciences University of Hull Hull UK

**Keywords:** citizen science, deer, disease, risk assessment, species distribution modeling, wildlife management

## Abstract

**Aim:**

Decisions on wildlife conservation, management, and epidemiological risk are best based on robust evidence. The continual improvement of species distributions, such that they can be relied upon in decision‐making, is important. Here we seek to refine aspects of a generic modelling approach and improve the utility of species distribution maps.

**Location:**

Great Britain (GB).

**Methods:**

We applied a modeling framework based on hierarchical Bayesian species distribution models exploiting opportunistic occurrence records from citizen science datasets to predict both current and potential distributions for each of the six deer species known to be present in GB. Using the resulting maps, we performed a simple analysis of the overlap between species to illustrate possible contact, which we interpret as the relative risk of potential disease spread given an introduction.

**Results:**

Predicted distribution maps showed good agreement with the broader scale occurrence reported by a recent national deer survey with an average True Skill Statistics and AUC of 0.69 and 0.89, respectively. Aggregation of the maps for all species highlighted regions of central and eastern England as well as parts of Scotland where extensive areas of range overlap could result in interspecific contact with consequences for risk assessments for diseases of deer. However, if populations are allowed to expand to their predicted potential, then areas of overlap, and therefore disease interspecific transmission risk, will become extensive and widespread across all of mainland Britain.

**Main conclusions:**

The generic modeling approach outlined performed well across all of the deer species tested, offering a robust and reliable tool through which current and potential animal distributions can be estimated and presented. Our application, intended to inform quantitative risk assessments, demonstrates the practical use of such outputs to generate the valuable evidence required to inform policy decisions on issues such as management strategy.

## 1 INTRODUCTION

The wise management of natural resources demands high‐quality information on which to base sound decisions (Regan et al., 2005). Fundamental to proportionate and evidence‐lead approaches to wildlife management are robust descriptions of where species are (their distribution) in the landscape. Increasingly, this can be achieved entirely through empirical surveys conducted by citizen scientists (McKinley et al., 2017). For example, the British Deer Society (BDS) distribution survey which, using information collected from members and other sources, has provided 100 km^2^ resolution distribution maps every five years since 2002 for each of the six deer species known to inhabit Great Britain (GB) (two native deer, red *Cervus elaphus* and roe *Capreolus capreolus*, one naturalized, Fallow *Dama dama* and three non‐native species, Chinese water deer *Hydropotes inermis*, Reeves’ Muntjac *Muntiacus reevesi* and Japanese sika *Cervus nippon*). The most recent update was published in 2018 (https://www.bds.org.uk/index.php/research/deer-distribution-survey) based on data collected between 2012 and 2016.

While informative for monitoring broad‐scale changes in species range (Ward, 2005), such coarse descriptions lack the finer detail we argue necessary to assess the potential intra‐ and interspecific interactions important for the accurate estimation of, for example, pathogen transmission risk between neighboring populations at an ecologically relevant scale (i.e., home range; Hartley, Voller, Murray, & Roberts, 2013). However, producing comprehensive fine‐scale descriptions (e.g., 1 km^2^ resolution; Croft, Chauvenet, & Smith, 2017) through exhaustive empirical survey alone, particularly across a national extent, rapidly becomes infeasible. In such cases, qualitative and quantitative analysis is required to produce the most robust inference possible from partial data which can then be used to inform predictions to fill data gaps.

Species distribution models (SDMs; Guisan & Zimmermann, 2000; Elith & Leathwick, 2009) such as MaxEnt (Phillips, Anderson, & Schapire, 2006) have become a popular solution, providing a formal quantitative process to relate the presence, and absence, of species against a set of environmental drivers, for example, climate, land cover, human interference, and topography, the understanding of which can be used to inform prediction of occurrence in unsurveyed locations. The widespread use of SDMs has been further accelerated by the proliferation of large publically available data repositories such as the National Biodiversity Network (NBN) Atlas, providing access to occurrence datasets, typically opportunistic sighting records, from a wide range of providers including local records centers, national enthusiast groups, wildlife charities, government and environmental consultants. Despite the popularity of SDMs, however, it is important to recognize that this is a “young,” rapidly developing science with many proposed methods, each based on subtly different sets of assumptions, but no consensus for a single unified framework (Croft et al., 2017; Croft, Smith, Acevedo, & Vicente, 2018). As such great care must be taken to avoid inappropriate application and incorrect inference (Guillera‐Arroita et al., 2015); a particular consideration for the work here which seeks to provide output more suitable for use in policy development and decision‐making in the context of current and future risks posed by wildlife disease to human interests. For instance, opportunistic occurrence data of the type collected through citizen science are known to be subject to reporting bias (Callcutt, Croft, & Smith, 2018; Dickinson, Zuckerberg, & Bonter, 2010) but is often ignored (Wheeler, Ward, Smith, Petrovan, & Croft, 2019). Similarly, a fundamental assumption of most SDMs is that species are at equilibrium with their environment, that is, where species occurrence can be explicitly described by environmental conditions, which cannot be assumed for many, particularly non‐native and heavily managed species, where absences (and potentially presence) may be dispersal‐limited or anthropogenically mediated (e.g., translocated to new locations, or hunted/ managed until absent at suitable locations). Again, this issue is often ignored (see Wheeler et al., 2019) but if not accounted for can seriously confound model predictions (Hattab et al., 2017).

In this paper, we outline a general methodology based on a hierarchical Bayesian modelling framework (Latimer, Wu, Gelfand, & Silander, 2006) to estimate both the realized (current) and potential (future) distribution of terrestrial mammal species using opportunistic occurrence data accounting for both reporting bias (detectability) (van Strien, Swaay, & Termaat, 2013) and critically dispersal limited/anthropogenic influences (Hattab et al., 2017). We assess the validity of this method considering an example case study focusing on British deer; for which good data are available both to fit models and to perform independent validation. Reports published over the past decade or so suggest that most of these populations have been steadily growing (Battersby, 2005; Mathews et al., 2018; Ward, 2005). In large numbers, deer can inflict substantial damage to woodland and crops (Putman & Moore, 1998) as well as providing a reservoir for the transmission of diseases, some of which can affect livestock and human health, for example, bovine tuberculosis (Ward & Smith, 2012) and foot‐and‐mouth disease (Böhm, White, Chambers, Smith, & Hutchings, 2007). In contrast, their populations may be threatened by the introduction of novel diseases, such as chronic wasting disease (Ricci et al., 2017) which has severely impacted some cervid species in the USA (Monello et al., 2014) and has recently been reported in Europe (Benestad, Mitchell, Simmons, Ytrehus, & Vikøren, 2016). This particular disease can be transmitted by both direct (nose‐to‐nose) and indirect contact through contamination of the environment (Mathiason et al., 2009; Plummer, Johnson, Chesney, Pedersen, & Samuel, 2018). With this in mind, we demonstrate the value of the national deer distribution estimates that we generate as a contribution to a future quantitative risk assessment for diseases that can be shared between multiple species, such chronic wasting disease. In this context, our maps can be used to inform policy development and decision‐making by exploring the current scale and location of range overlap (potential contact) between species, as well as the potential extent of their future overlap (once all species have reached equilibrium), which could be interpreted as a future pattern of relative risk of interspecific disease spread across the country.

## 2 METHODS

### 2.1 Study area

In this study, we chose to limit the model extent to mainland Great Britain (219,536 km^2^). For terrestrial mammals, substantial channels of water can act as a natural barrier prohibiting, or substantially reducing, regular movement, or dispersal. While some deer are known to cross open water in some contexts, their effective dispersal and establishment on islands is better represented on broader geological timescales and is not relevant here. To reflect this, we only considered a cell to be connected to the mainland if it occurred in the Moore neighborhood (eight directly adjacent cells) of the mainland on a 1 km^2^ raster of the British National Grid (BNG).

### 2.2 Occurrence data

Occurrence data were downloaded from the NBN Atlas on 13/09/2018. We restricted our download to mammal observations (direct or indirect evidence of presence described with coordinates and corresponding precision, date and taxonomic description) recorded between 2012 and 2016 (the same period as the recent national deer survey undertaken by the British Deer Society) from two national datasets only: The Mammal Atlas Project provided by The Mammal Society (https://doi.org/10.15468/i2eosa) and BTO nonavian taxa provided by the British Trust for Ornithology (https://doi.org/10.15468/2m9nxa). Several factors contributed to our decision to restrict the download to national datasets but primarily we argue it maintains greater consistency in recording effort across regions and reduces the possibility of duplicate records across datasets. Any records without an exact sighting date, taxonomic description to the species level and coordinate accuracy equivalent to or better than the 1 km^2^ BNG, or whose location lay outside mainland Britain were excluded.

One of the limitations of opportunistic observations of the type generated from citizen science, comprising a large proportion of the records downloaded, is the lack of information regarding survey effort. This is important in understanding whether the absence of data, in this case presence records, is evidence of true absence of a species or merely insufficient effort to detect it. Previous studies (Croft et al., 2018; Phillips et al., 2009; van Strien et al., 2013) have suggested that records of other species may provide a suitable proxy to estimate survey effort. Here, we considered records of other deer species together with common mammals easily identifiable by a similar method of visual observation alone (both direct and indirect, e.g., including evidence of species presence such as burrows, mounds and scat). Specifically, we considered records of fox (*Vulpes vulpes*), gray squirrel (*Sciurus carolinensis*), rabbit (*Oryctolagus cuniculus*), hare (*Lepus europaeus*), mole (*Talpa europaea*), rat (*Rattus norvegicus*), and cat (*Felis catus*); see Croft and Smith (2019) for details. Using these records, we computed binomial datasets for each of the deer species describing the number of successes as individual visits (unique 1 km^2^ BNG cell and date) where the target species was reported and the number of trials as visits where any of the species considered, including the target species, was reported. The aim of representing the data in this way was to provide information not only in terms of presence but also observability and by association likelihood that nondetection of a species was an indication of true absence or not, in that we are able to determine whether sufficient visits occurred to be confident that if a species were present it would have been reported.

### 2.3 Explanatory variables

Following Acevedo, Ward, Real, and Smith (2010), we considered a range of environmental factors that might influence British deer distributions, including descriptions of climate (temperature and precipitation: Fick & Hijmans, 2017), topography (altitude and slope: OST50 www.ordnancesurvey.co.uk/business-and-government/products/terrain-50.html), human disturbance (distance to roads and urbanization: OS Strategi www.ordnancesurvey.co.uk/business-and-government/products/strategi.html and Rowland et al., 2017), and habitat structure (area of mixed broadleaf and coniferous woodland, arable land, pasture, upland, inland rock, freshwater, and supra‐littoral rock; Rowland et al., 2017).

Co‐correlation between environmental factors, for example, climate variables with very similar spatial patterns, was minimized by transforming our set of environmental factors, based on values extracted from the assumed range of each species (defined in the next paragraphs), using the “prcomp” function in R applying variable scaling to perform a principal component analysis (PCA) and to inform a new set of independent, linearly uncorrelated variables ordered according to their contribution toward explanatory variance. We elected to retain all of these new variables (the same number as the original set of factors; 24 in total) rather than seeking a reduction based on explained variance (Croft et al., 2018) which is sometimes applied to simplify models, avoid overfitting or to provide insight into the main drivers of a species' distribution. This was not deemed necessary here as all variables were shown to contribute a nonzero proportion of the explained variance, there were no computational constraints to force a reduction, and biological insight was not an objective of this study. However, reduction may be necessary in the future if additional variables were to be considered.

In addition to the environmental variables common to most species distribution models, we also considered a spatial factor, to help account for presence observations at locations where a species might otherwise not be expected able to survive unassisted (i.e., observations of animals produced by anthropogenic translocation or maintenance), or absences from areas unexplained by the environment, and also likely to be caused by man (e.g., local or regional scale hunting or persecution, and the gaps between establishing populations of non‐native species). This factor allowed us to leverage the information entailed in the broad‐scale description of a species within its range and account for observations which express a temporal, geographical, and anthropogenic deviation from the natural dispersal and persistence of species (Hattab et al., 2017). Inclusion of such a variable is an important but often overlooked concept in modeling both current and potential distributions. Firstly, it produces a data‐driven description of the extent from which to select absence locations. It also provides a mechanism to mitigate failure in the assumption of equilibrium across the extent and explain current absence in environmentally suitable but spatially independent locations. Maps of the variation in this factor will also indicate areas for which new observations are less valuable (well within the range of a well described species) or are more valuable (in specific areas of a range edge, or in combinations of poorly represented (sampled) environmental factors.

Several methods have been proposed to estimate species range (extent of occurrence) from sightings data using global or, more recently, local bounding geometry (Maes et al., 2015). The latter local methods, such as localized convex hulls (LoCoHs), are generally recognized as providing more realistic estimates than their global equivalents, minimizing influence from the most extreme points which can lead to the inclusion of large areas of unsuitable or untested environments (Burgmann & Fox, 2003). LoCoHs define a species’ range as the union of a set of “localized” minimum convex hulls (MCHs) fitted to subsets of the data. These local subsets or neighborhoods, one for each “root” point within the global dataset, can be determined according to various criteria using, for example, a fixed number of, or maximum distance to, neighboring points. It is suggested by Maes et al. (2015) that criteria based on distance are most robust to the sporadic but spatially clustered recording commonly featured in opportunistic citizen science data.

While conceptually LoCoH provides a good option to estimate species range, in this study we applied a similar but arguably simpler, more inclusive approach extending the idea to nominally reflect the potential dispersal range of each species from their known occurrences in an attempt to compensate for imperfect detection. For each positive sighting location (cell with at least one recorded observation of the species), we defined a circular local neighborhood similar to the distance applied in LoCoH (Mathews et al., 2018). Rather than using all other positive sightings locations (points) within this “sphere of influence” to create a MCH, we simply overlay all of the neighborhoods and count the number of intersections. In order to account for the likelihood that some sightings may lie on the edge of the species’ range, we threshold the resulting map, only retaining cells intersecting three or more neighborhoods. Cells considered to be within the species’ range were assigned a value 1 with cell values outside of this range assigned values decreasing to zero exponentially, at the distance equivalent to that defining a neighborhood from the estimated species’ range. This helped to smooth the transition at the range edge and reflects the possibility that longer distance dispersal may have occurred but has not yet been recorded.

It should be noted that the precise distance chosen to define a neighborhood is a complex parameter and is the combination of multiple factors including daily or seasonal mobility, dispersal, as well as the aims of the study, that is, whether to be inclusive and include more unsuitable environments (commission errors) or more exclusive and risk omitting suitable environments (omission errors) and the spatial coverage of the data (Maes et al., 2015). To establish the most suitable distance for each species, we compared model performance (predictive accuracy as described later) testing increasingly inclusive species ranges with neighborhood distances between 10 km and 100 km. This range was chosen to encompass the magnitude of distances used in other studies (Maes et al., 2015; Mathews et al., 2018) and the known limits of species dispersal (e.g., Hartley et al., 2013).

### 2.4 Model

We modeled the current probability of occurrence (and subsequently potential probability of occurrence analogous to environmental suitability) for each species using the “hSDM.ZIB.iCAR” function of the “hSDM” package (Vieilledent et al., 2014) in R statistical software (R Development Core Team, 2018) applying default settings except to reduce the number of iterations to 1,500 (500 for burn‐in and 1,000 for sampling) and the thinning interval to 1. This function used our binomial dataset (presences/successes and visits/trials) within a hierarchical Bayesian framework integrating two processes: (a) an ecological process, represented by a Bernoulli distribution, describing species presence or absence due to environmental suitability; (b) an observation process, represented by a binomial distribution, which takes into account the fact that detection of the species is imperfect (i.e., likely to be <1) (Latimer et al., 2006; MacKenzie et al., 2002). The ecological process included an intrinsic conditional autoregressive (iCAR) model for spatial autocorrelation between observations, assuming that the probability of presence of the species at one site depends on the probability of presence of the species on neighboring sites (Lee, 2013). For the purposes of this study, we applied a Moore neighborhood representing the maximum daily home range for any of the deer species. In this study, due to limitations on the volumes of available data, we did not account for any temporal variability in species distribution within the window of interest, 2012–2016, which we suggest was sufficiently narrow to justify this simplification (i.e., that distributions remain effectively stable within the period).

Modeling the ecological process, we considered the full set of explanatory variables including our estimation of current species range (dispersal). Whereas for the observation process we only considered a constant to reflect that the number of trials was derived from other presence records whose detectability within any given cell was likely to be similarly affected by the environmental condition; therefore, detectability was represented in relative terms compared to that of other species (i.e., difference in average size or general behavior of species rather than environment). For all model parameters (coefficients) in both ecological and observation processes, we used default uninformative Normal priors with a mean of zero and large variance of 1e^+06^ providing a relatively flat distribution. Similarly, we used the default uninformative prior for the variance of the spatial effects; described by an inverse‐gamma distribution with shape and rate parameters of 0.05 and 0.0005, respectively.

**Figure 1:**
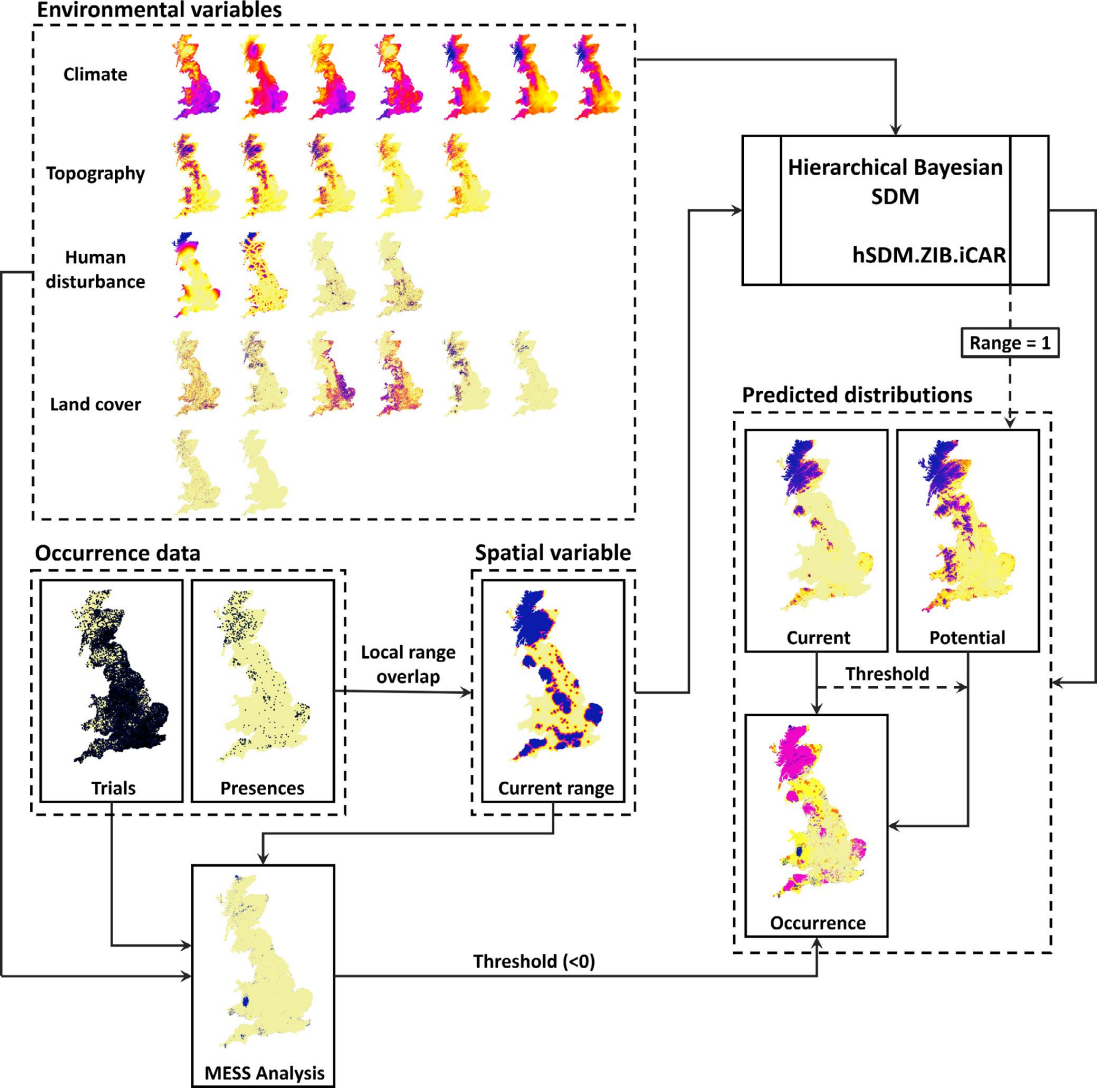
Schematic diagram of the modelling process

Once fitted, we first used the model to extrapolate the probability of species occurrence across the entire model extent based on all variables including those describing species ranges to produce current distributions, and then, setting species range to a constant value of 1 we produced a second set of distributions reflecting the potential of each species based on environmental conditions alone assuming no constraints on dispersal. A schematic summarizing the complete modeling process is provided in Figure 1.

### 2.5 Validation

In order to validate our predictions of current deer distributions, we undertook evaluation using independent data obtained from the recent BDS deer survey. This national survey provides the presence, and reported absence, of deer across GB on the 10 km^2^ BNG. To match the resolution of this dataset, we aggregated our 1 km^2^ suitability maps assigning the maximum probability of presence to each 100 km^2^ cell. Based on these datasets, we computed two standard indices to measure goodness of fit: the area under the receiver‐operating characteristic curve (AUC; Phillips et al., 2006) yielding a value between 0.5 and 1 where 0.5 suggests models no better than random and 1 indicating perfect prediction; the True Skill Statistic (TSS; Allouche, Tsoar, & Kadmon, 2006) calculated as the sum of the sensitivity (true positive rate) and specificity (true negative rate) minus one, yielding values between 0 and 1 where similar to AUC a value of 1 indicates perfect prediction. The latter requires a binary classification (presence/absence) for both the observed and predicted datasets. To transform our predictions into presences and absences, we selected the threshold probability above which presence (and equal to or below absence) is assumed which maximizes the TSS (Liu, White, & Newell, 2013).

While this threshold is applicable to classify presence and absence for the current distributions, it is not necessarily appropriate to classify our potential distributions, which are derived under different conditions. In order to provide an equivalent threshold for these distributions, without comparable data (which are of course unavailable), we adopted the minimum probability of presence extracted from cells classified as present in the current distribution (which we assume will continue to be occupied in the future) and that are also within the current species range (i.e., where the dispersal variable equals 1 in models for both the current and potential distribution).

Finally, to evaluate the limits of our predictions based on the available presence data, accounting for any impacts from preferential sampling common in data from citizen science (Callcutt et al., 2018) as well as the possibility that not all species have experienced the entire range of environmental conditions and hence their response is unknown, we computed a multivariate environmental similarity surface (MESS) provided in the “dismo” R package. This function compares the differences between environmental ranges sampled in the model training data (within the assumed current range of each species) with that used to project distributions across the entire model extent (Elith, Kearney, & Phillips, 2010). In the resulting map, outputs cells with negative values (<0) indicate environmental conditions that are not well represented in the model training data, that is, the model has little to no experience of dealing with the related values and consequently any predictions may not be valid.

### 2.6 Local range overlap

Frequency of contact (direct or indirect), or exposure, between suitable hosts is an important factor in the spread of disease. While the resolution of modelled output here is not sufficient to establish the precise “connectedness” of British deer populations as it does not account for physical barriers that may preclude interaction, we suggest that the maps provide sufficient information to examine a “worst case” scenario, assuming such barriers are not absolute, using local range overlap as a proxy for relative disease risk. Local range overlap was computed by combing predictions for current and potential distributions as follows: for each cell, we estimated contact as the area spanned by the contiguous region surrounding the target cell formed by the overlapping distributions of those deer species present in that cell. In the context of disease risk, this combined measure of intra‐ and interspecies contact can be considered analogous to the area of immediate exposure following an introduction in the target cell.

## 3 RESULTS AND ANALYSIS

### 3.1 Occurrence data

Following cleaning and transformation to a binomial description, the dataset of observational records comprised of 660,107 distinct trials (unique day, location and dataset) distributed relatively evenly across 24,844 cells throughout the model extent (approximately 10% of mainland Britain). These trials were derived from the records of common mammal species including all deer species and represent the frame from which observed presences and absences could reliably be inferred. Roe deer were reported most frequently with 11,605 unique sightings spread across 4,690 cells. Interestingly, muntjac were the next most commonly reported with 7,591 sightings across 927 cells (the highest density of unique sightings per cell) followed by fallow (2,422 sightings over 1,820 cells), red (1,340 sightings over 760 cells), sika (267 sightings over 157 cells), and then Chinese water deer limited to 185 unique sightings across 114 cells.

### 3.2 Species range estimation and validation

**Figure 2:**
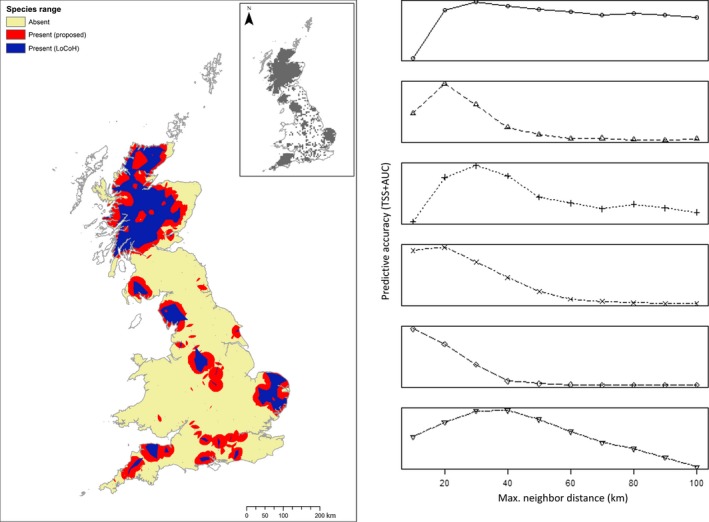
Estimating species range. (Left) Example map showing estimate range for red deer from opportunistic occurrence data using Local Convex Hulls (blue) and our method of overlapping neighborhoods (red). (Right) Plots showing measured predictive accuracy of models afvgainst maximum distance used to define neighborhoods in derivations of species range for (top to bottom) Chinese water deer, fallow, muntjac, red, roe, and sika

A visual comparison between estimated species ranges generated using both our method and LoCoH (illustrated in Figure 2) showed similar results but as expected confirmed our method to be more inclusive. Additional inspection against the latest distributions from the BDS survey showed that this greater inclusivity provided better agreement.

With regard to the choice of neighborhood distance, and therefore the inclusivity of the species range, our findings shown in Figure 2 demonstrate that the predictive accuracy of models can varied markedly dependent on the distance assumed. In these plots, peaks where the combined average of AUC (rescaled from between 0.5 and 1 to between 0 and 1) and TSS is highest, indicate optimal neighborhood distance. All species demonstrated a clear optimum between 10 and 40 km (30, 20, 30, 20, 10 and 40 km for Chinese water deer, fallow, muntjac, red, roe, and sika, respectively) with predictive accuracy of models using distances beyond this upper limit rapidly decreasing. At their optimal distance, used to predict final distributions, model AUC for all species was well above the 0.7 threshold considered to be indicative of a good predictive accuracy (Hijmans, 2012) with values of 0.95, 0.82, 0.95, 0.87, 0.9, and 0.84 for Chinese water deer, fallow, muntjac, red, roe, and sika, respectively. Across these species, of the distances considered 10, 20, and 30 km maintained AUC scores above the 0.7 threshold for all with a minimum value of 0.77, 0.82, and 0.71, respectively.

### 3.3 Predicted distribution

**Figure 3:**
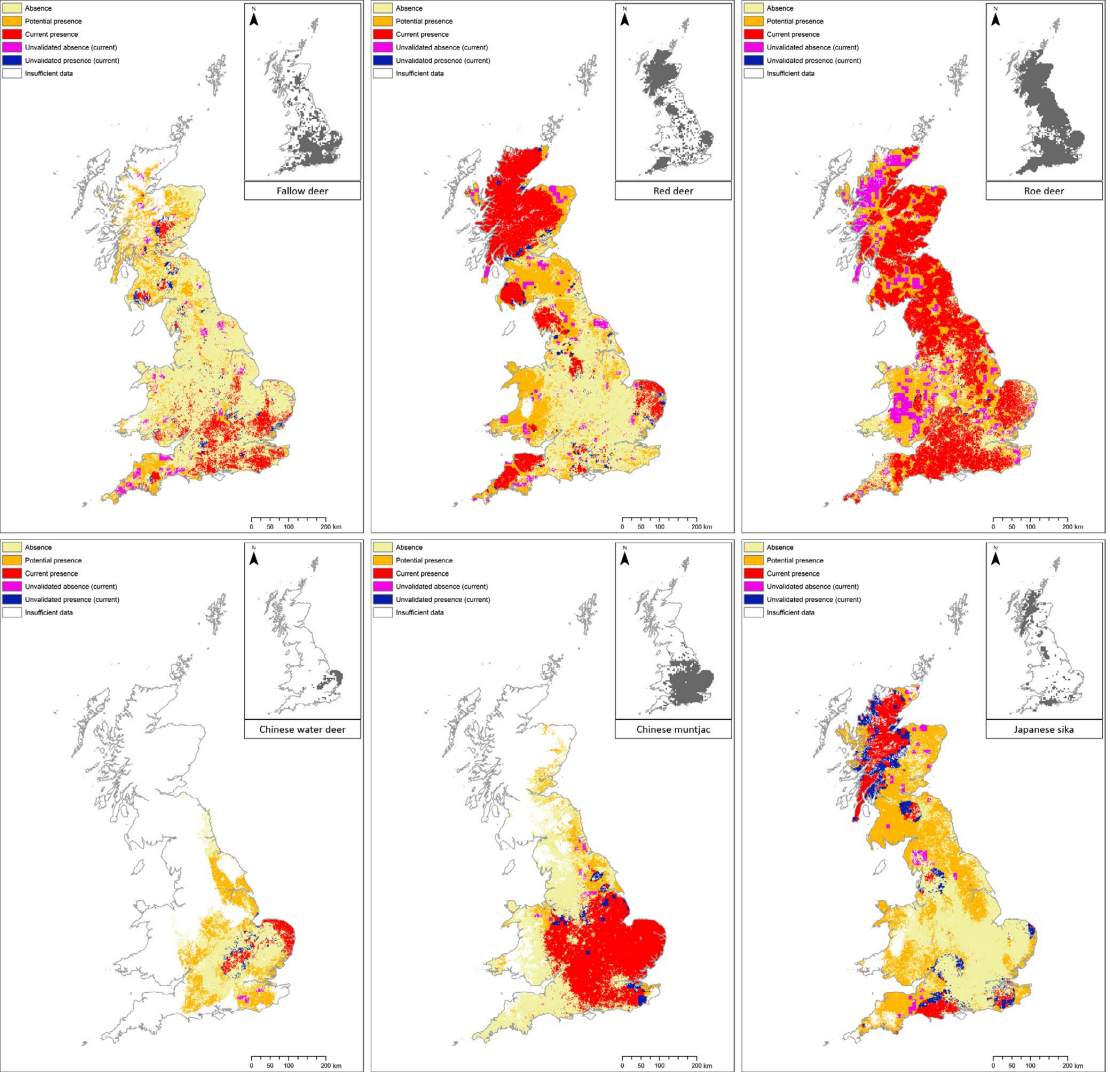
Current and potential deer distributions. Maps show predictions for both the current distribution, accounting for anthropogenic and geographic factors that might prevent presence (red), and potential expansion based on environmental factors alone ignoring any limitations on dispersal (orange) for each of the six deer species in GB. Within each map, we highlight locations where predictions are uncertain either because conditions are beyond that experienced by the species (and so it unknowable how they will respond) or predictions are not validated by the corresponding BDS survey map (inset) and therefore require further investigation

Our descriptions of the current and potential distribution of each deer species (Figure 3) allowed us to highlight locations requiring further survey effort where either the environmental conditions were not well represented by the current survey (potentially as the species may have yet to experience them and so it would not be appropriate to assume the response) as determined from the MESS analysis, or the prediction was not validated by the latest BDS deer survey. Occupancy statistics summarizing the distributions shown in these maps are provided in Table 1.

**Table 1:** Summary of current and potential occupancy statistics for each of the six deer species

Statistic	CWD	Fallow	Muntjac	Red	Roe	Sika
Current range (km^2^)	16,269	76,166	85,068	75,998	118,186	52,786
Current occupancy (km^2^)	6,106	24,502	62,482	59,155	101,852	34,596
Occupancy (%)	37.5	32.2	73.4	77.8	86.2	65.5
Potential occupancy (km^2^)	29,215	59,514	76,129	121,081	174,763	116,402
Growth (%)	378.5	142.9	21.8	104.7	71.6	236.5
Unsampled (%)	70.0	13.6	34.3	2.3	2.4	8.2

Note

Statistics include the area spanned by the estimated current range and distribution with corresponding percentage occupancy; the area spanned by the predicted potential distribution and the percentage increase (growth) relative to the current distribution; and the percentage of the total model extent (spanning 219,536 km^2^) not represented by the environments sampled within the species' current range.

National projections for Chinese water deer are difficult due to their limited experience of the British landscape, only providing a narrow sample of the environment (corresponding to approximately 30% coverage of the model extent) with which to infer environmental preferences. Nevertheless, even at a local level, comparison of their current distribution with their potential distribution suggests the species may be reaching local equilibrium but with potentially suitable environments within reach, just outside of range edges. Thus, expansion could continue allowing exploration into new, as yet untested environments toward the northwest and southeast of England; potentially increasing occupancy from the current 6,000 km^2^ to 30,000 km^2^ (growth of nearly 400%). Muntjac, to a lesser extent, present a similar challenge with current distributions only representing a limited sample of environmental conditions; spanning 62,500 km^2^ but only providing a representative environmental sample for 65% of the model extent. Nevertheless, excluding the northwest of Scotland, our predictions suggest that the muntjac is beginning to reach the full extent of its potential range within Britain, with only limited scope for further expansion up the northeastern coast of England and some small isolated patches in Wales, a maximum increase in occupancy of 22%.

Comparing distributions for red, roe, and sika suggests the greatest potential for further expansion is into Wales where current occupancy is low given its extensive area of apparently favorable environment; recent reports of increasing populations support this prediction (Mathews et al., 2018). Both red and sika share similar potential distributions spanning 121,000 and 116,000 km^2^, respectively, with suitable environments across most of Scotland, Wales and the southwest of England. Predictions for roe, which already occupy much of England and Scotland (predictions from their current distribution suggest 50% by area), highlight only a few areas where the species would not be expected to eventually establish due to environmental unsuitability; approximately 18% of the total model extent. Fallow distributions appear relatively patchy in comparison with other species. Current populations are focused in central and southern England with a few isolated populations in Scotland and the south west, which show the greatest potential for future expansion. Overall for fallow, we predict a potential increase in occupancy of 143% from the current distribution spanning 25,000 km^2^ to 60,000 km^2^.

Across all of the species, our results highlight several areas where the attention of citizen scientist might most profitably be focused to confirm, or deny, presence, including the north of Scotland. It is interesting to note that un‐validated predictions tended to appear at the edge of patches, which is likely a reflection of the accuracy of our estimates for species’ range.

### 3.4 Local range overlap

**Figure 4:**
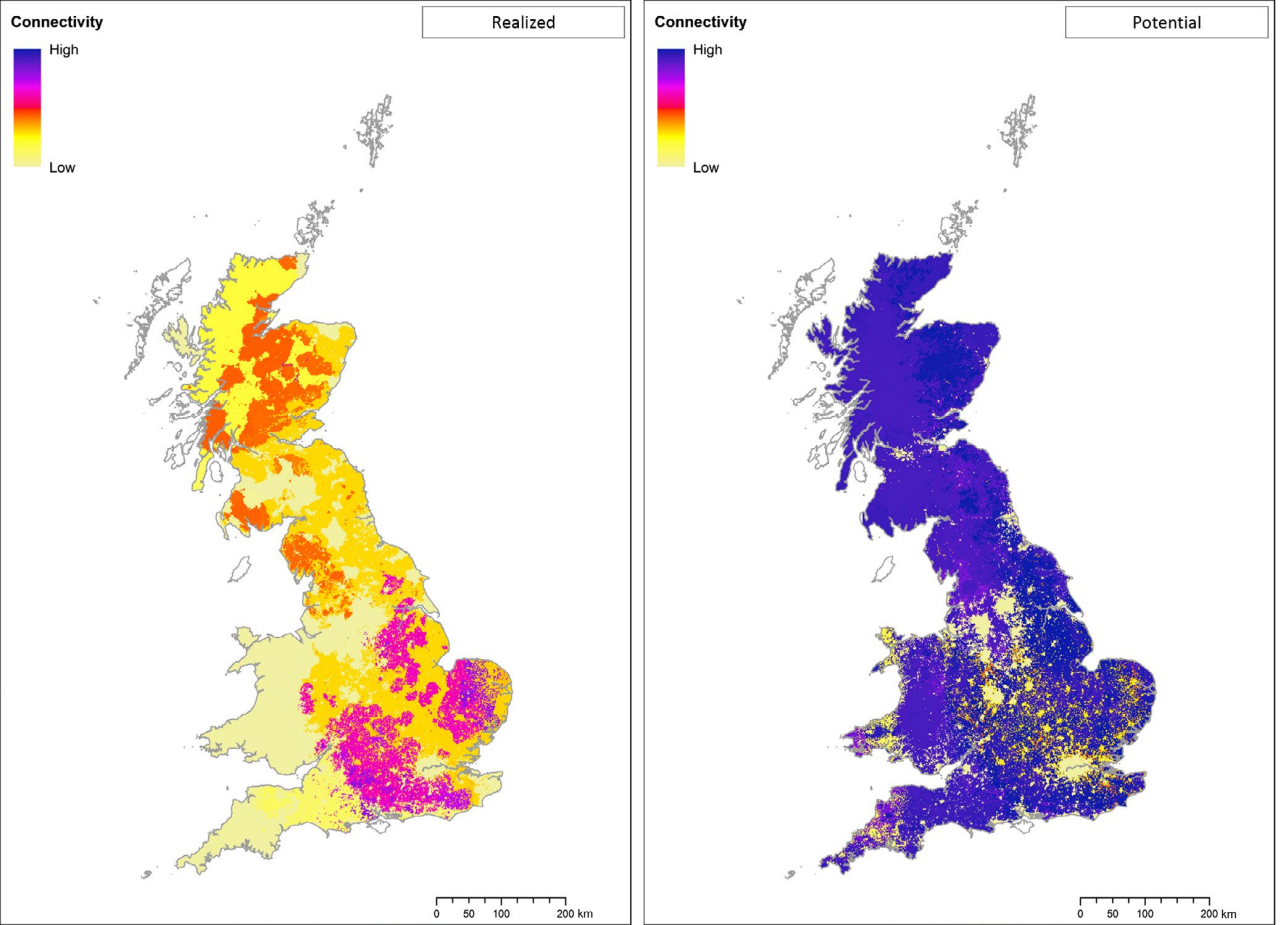
Estimated levels of contact between British deer populations. Maps showing current (left) and potential (right) show the immediate extent of contact between deer in cells (quantified as the area in km^2^ formed by the contiguous local deer population, excluding effects from long‐distance dispersal, of those species predicted to occur in the cell) to visualize the risk posed by disease introduction. Darker, more intense, colors reflect greater contact and therefore are assumed to present greater risk

Combining the estimates of the ranges of the current distributions predicted by the suitability models across all species indicates potential for high level of contact (Figure 4) across deer populations in southern central and eastern regions of England, followed by areas of Scotland and the Lake District (this represents the degree and area of overlap between multiple species and contiguity of deer in the landscape). The similar exercise using potential distributions shows that if all deer species spread to their full potential and achieve equilibrium in the landscape, contact will likely increase substantially across the country becoming notably more uniform, that is, potential for very high levels of contact between deer across the entire extent of Britain (Figure 4).

## 4 DISCUSSION

Here, we have proposed a generic framework using an SDM approach to estimate both the current and potential distributions of mammals; a valuable resource to inform decisions on the management of current and future disease risk. In addition to allowing us to begin to accommodate problems caused by dispersal‐limited/anthropogenic‐driven absences, this method also incorporates mechanisms to account for the types of survey bias known to occur in citizen science data (Callcutt et al., 2018; Dickinson et al., 2010). We tested the method using British deer species as a case study for which recently published survey data from the BDS were available to provide independent validation. This validation indicated that the method performed well for all species, achieving an AUC value greater than 0.7, the accepted threshold indicating good predictive accuracy, based on at least one of the underlying species' ranges that were tested (derived using different distances to define a local neighborhood connecting nearby observations). A comparison of validation metrics with those from previous studies (Acevedo et al., 2010; Croft et al., 2017) which do not explicitly account for either survey bias or nonequilibrium of species suggests that our proposed method produces more accurate results. Acevedo et al. (2010) reported AUC values of 0.85, 0.79, 0.86, 0.82, 0.93, and 0.93 for roe, red, fallow, sika, muntjac, and Chinese water deer, respectively. Compared with values here of 0.9, 0.87, 0.82, 0.84, 0.95, and 0.95, respectively, all were higher with the exception of the model for fallow deer which was marginally lower. Compared with Croft et al. (2017) where AUC scores for models at a 1 km^2^ resolution were reported as 0.64, 0.63, 0.65, 0.64, 0.66, and 0.68, respectively, there is a marked improvement in predictive accuracy. It should be noted, however, unlike Croft et al. (2017) where both modelling and validation are conducted at a 1 km^2^ resolution, the model here was validated using data recorded at 100 km^2^ following an upscaling of the model output. As a consequence, validation metrics may be more indicative of the fit to species range rather than finer scale distribution. Further research is required to establish how such models, in the absence of true absence, can be reliably evaluated using available methods. Nevertheless, comparing instead results reported by Croft et al., 2017 for their models conducted at an equivalent 100 km^2^ resolution AUC values were similarly lower, between 0.7 and 0.76.

Unlike many other mammal species for which suitable data are unavailable, independent validation of our predictions for deer has allowed us to assess optimal neighborhood distances used to estimate species range. Our results show that a fixed distance of 20 km maintains the highest AUC scores across all deer species, above the 0.7 benchmark, and therefore, we argue might reasonably be applied more generally to estimate species ranges of diverse mammals without significant losses in model performance. Interestingly, this is the same distance adopted by The Mammal Society used to derive species ranges in their recent review of British mammals (Mathews et al., 2018). The only caveat to this is to note that optimal distances are based on several factors including mobility and patterns of recording (Maes et al., 2015); the latter likely explains the greater optimal distances for Chinese water deer and muntjac which, based on ecology alone, would otherwise be expected to be among those with the smallest dispersal neighborhoods. We would consider few British mammals to be more mobile than deer and so as an upper limit, the choice of 20 km as a distance for defining neighboring sightings is only likely to be too small for rare or poorly recorded species where the granularity of observations is low. This effect is seen in our results with the rarer invasive non‐native deer species showing highest predictive accuracy at greater distances despite evidence to suggest at least some range over smaller areas than the better recorded native species (Chapman, Claydon, Claydon, Forde, & Harris, 1993); in general for common species, this issue should not be a problem. A neighboring distance of 20 km may not be appropriate for small mammals such as rodents, but is likely to be suitable for larger mammals.

Visual comparison between predictions of current distributions based on available opportunistic survey data and observations from the more structured BDS survey shows good agreement. An important feature of the model framework and the maps we present for use by policy‐makers is the highlighting of areas where additional data are required to achieve sufficient predictive confidence. In this case, for well reported species such as deer in GB, few regions are highlighted as requiring further investigation either to establish absence where predictions suggest species should be present or vice versa. For some species (Chinese water deer and muntjac), predictions are restricted, we assume, by a lack of exposure to certain environments as a result of their relatively recent introduction and their current limited distributions rather than deficient sampling. Where this is the case for species naturally occupying the same bioclimatic zone, it may be possible to consider supplementing data, and corresponding inference of environmental dependence, from other locations where the species is present (Hattab et al., 2017). However, since Chinese water deer and muntjac both have warm temperate/subtropical origins, it is unlikely that environmental data from their native ranges will translate to the British landscape and it is perhaps most prudent to wait until such time as there are sufficient data for their invaded range.

Predictions for the potential distribution show some interesting results. In particular, highlighting that current distributions only reflects a fraction of the total extent available to most of the deer species. Even roe deer which already occupy much of England and Wales shows substantial potential for expansion into Wales. Combining these distributions to establish the potential extent and location of potential interspecific contact among deer suggests that unregulated range expansion could result in extensive and widespread areas of contact across the country. Under these circumstances, the rate of spread of diseases that affect multiple deer species (Hartley et al., 2013) and their geographical spread could be much greater than at present. If populations are more carefully managed, then fragmented distributions of some species might be maintained, limiting or even preventing transmission beyond the local area of introduction.

The application of our model outputs in this way to analyze disease spread illustrates just one of its many potential uses of policy interest across a host of concerns including wildlife conservation, management, and risk assessment. Our findings demonstrate that the modelling framework we have outlined and applied to British deer provides a generic tool capable of exploiting growing volumes of available citizen science data to generate useful information about species distributions in managed landscapes; importantly, highlighting areas where additional survey efforts are required to improve confidence in prediction. Distinct from many approaches in the literature, the framework accounts for both survey bias and dispersal/anthropogenic absences providing insights which together with clear presentation of where confidence in model predictions may be lacking, we argue are sufficiently robust to support policy decision‐making.

## CONFLICT OF INTEREST

None declared.

## AUTHOR CONTRIBUTIONS

S.C. conceived the main research idea under the guidance of G.C.S. and A.I.W. S.C. collated the data, designed the methodology with contributions from J.N.A. and led the manuscript writing. All authors contributed critically to the analysis and interpretation of the results and to the final draft of the manuscript.

## Data Availability

*Occurrence data*: Available from NBN Atlas (http://www.nbnatlas.org). *Environmental data*: (climate) Available from worldclim (http://worldclim.org); (topography) available from Ordnance Survey Terrain 50 (https://www.ordnancesurvey.co.uk); (land cover) available from CEH (https://eip.ceh.ac.uk) https://doi.org/10.5285/fdf8c8d3-5998-45a5-8431-7f5e6302fc32; (human population) available from Office for National Statistics and National Records of Scotland https://doi.org/10.5257/census/aggregate-2011-1; (roads) available from Ordnance Survey Strategi (https://www.ordnancesurvey.co.uk). *R scripts*: Available on request. *Study output*: Summary maps uploaded as online Supporting Information; raw data available on request.
