# Supplementary figures and images for: Modeling current and potential distributions of mammal species using presence‐only data: A case study on British deer
Source: Ecol Evol. 2019 Jul 11;9(15):8724–35. doi: 10.1002/ece3.5424 (PMC6686353; doi:10.1002/ece3.5424)

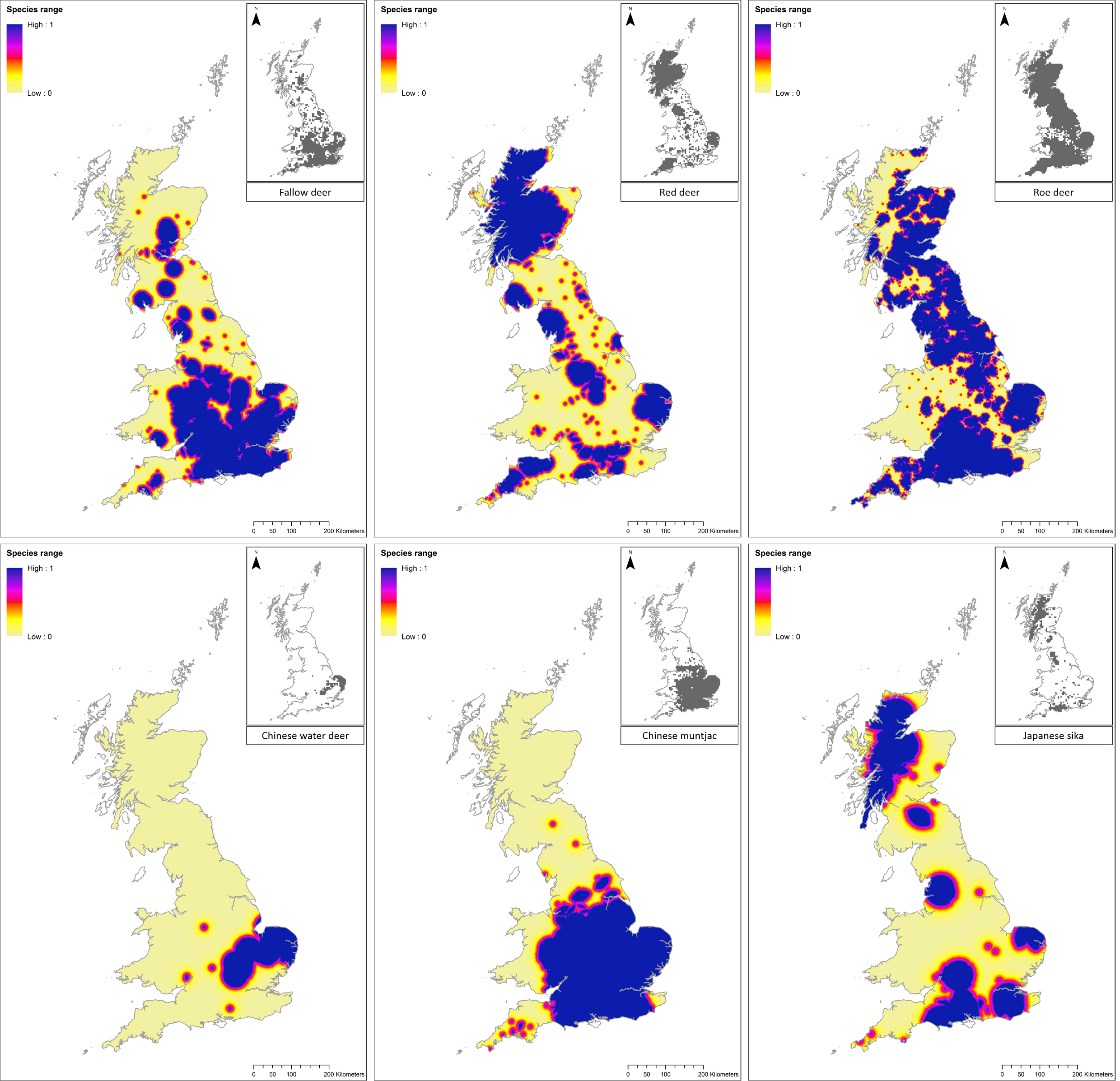

Supplement: Supplementary file 1 [file ECE3-9-8724-s001.tif]

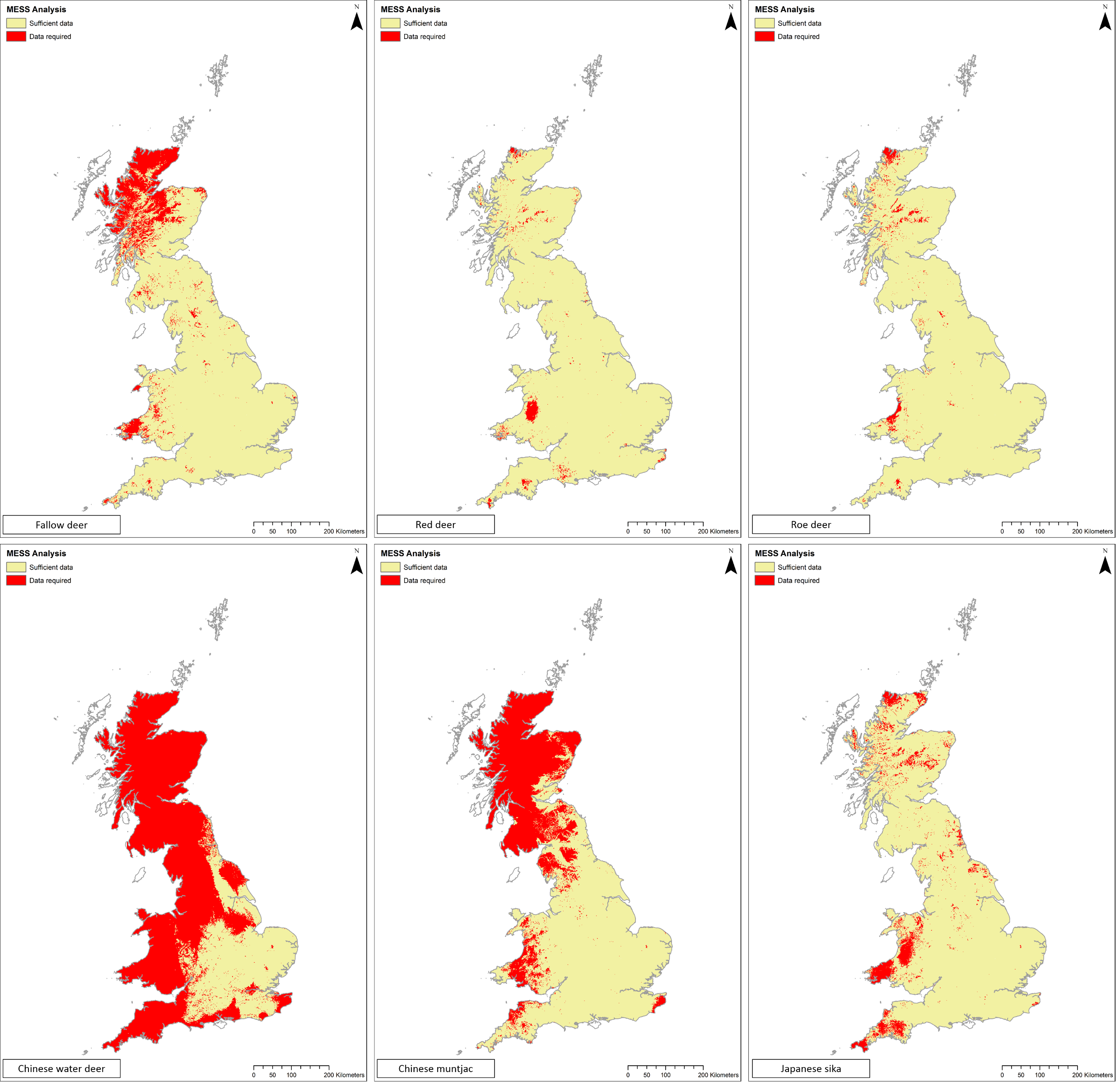

Supplement: Supplementary file 2 [file ECE3-9-8724-s002.tif]

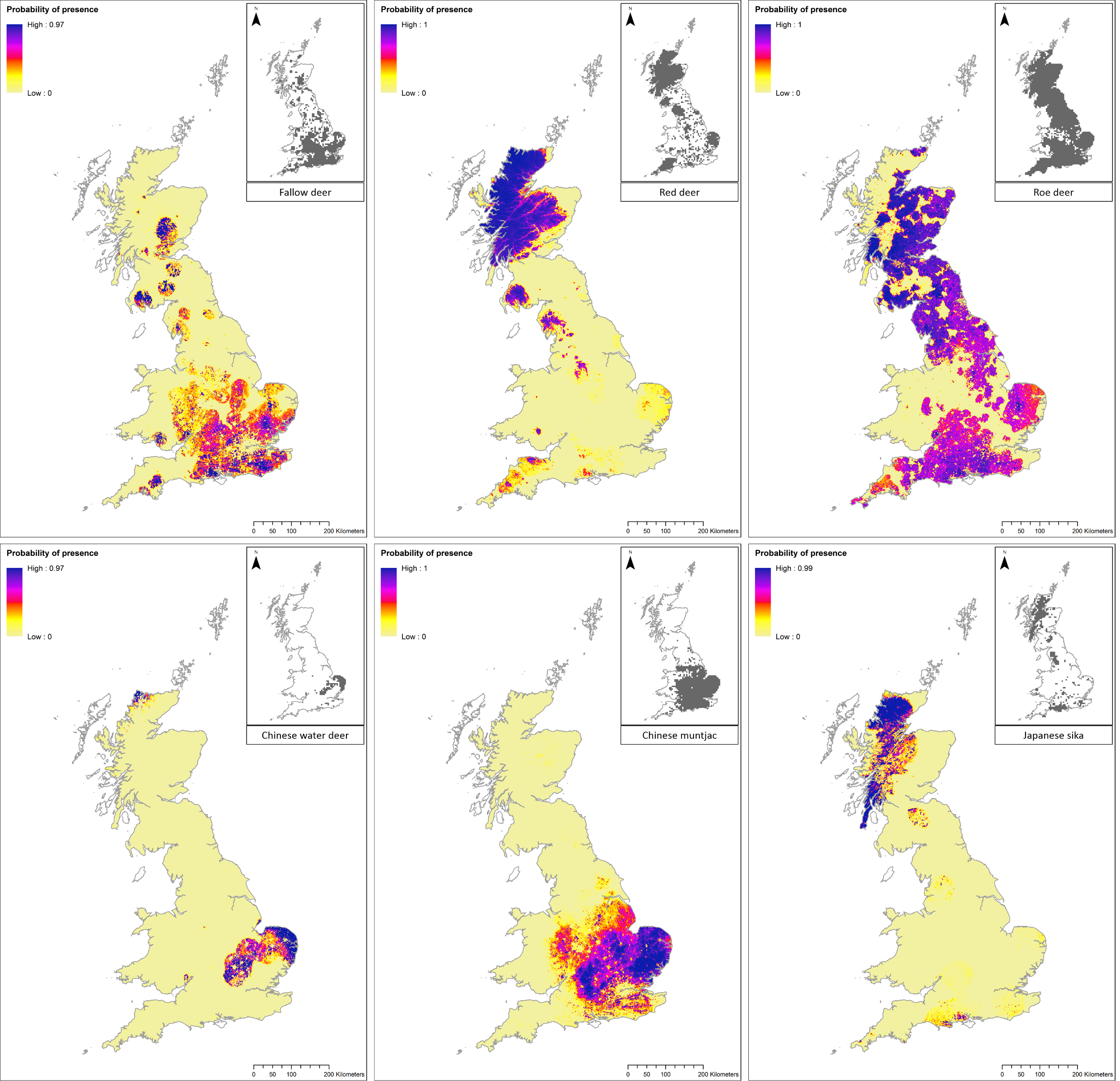

Supplement: Supplementary file 3 [file ECE3-9-8724-s003.tif]

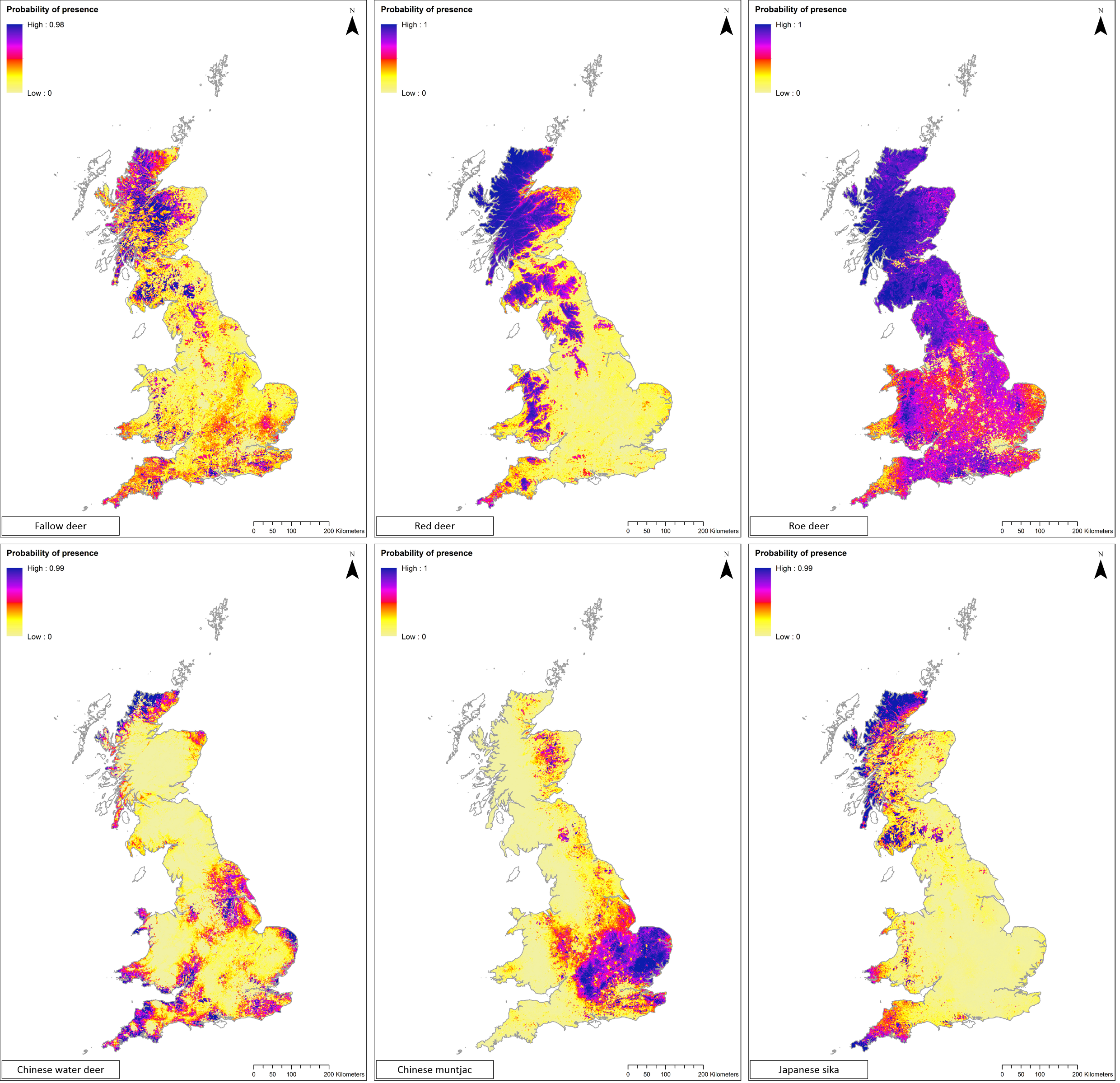

Supplement: Supplementary file 4 [file ECE3-9-8724-s004.tif]
