# Supplementary material for: Modeling current and potential distributions of mammal species using presence‐only data: A case study on British deer
Source: Ecol Evol. 2019 Jul 11;9(15):8724–35. doi: 10.1002/ece3.5424 (PMC6686353; doi:10.1002/ece3.5424)
Supplement: Supplementary file 5 [file ECE3-9-8724-s005.docx]

**Supporting information**

**S1 Figure: Species range (.tif).** Maps showing estimated range for each of the six deer species used in models.

**S2 Figure: MESS analysis (.tif).** Maps showing results of the MESS analysis for each of the six deer species classified to indicate where environmental conditions were (and were not) sufficiently represented by the model training data.

**S3 Figure: Current presences (.tif):** Maps showing predictions for the current probability of presence for each of the six deer species accounting for anthropogenic interference and limited dispersal.

**S4 Figure: Potential presences (.tif):** Maps showing predictions for the potential probability of presence for each of the six deer species based on environmental conditions alone assuming no anthropogenic effects or barriers to movement.
